# Variation of and associations with the depth and evenness of sequencing coverage in archived plastid genomes

**DOI:** 10.21203/rs.3.rs-5784537/v1

**Published:** 2025-07-14

**Authors:** Nils Jenke, Gregory M. Smith, Buddha Thapa Magar, Michael Gruenstaeudl

**Affiliations:** 1 Freie Universität Berlin, Institut für Bioinformatik, Berlin, 14195, Germany; 2 Fort Hays State University, Department of Computer Science, Hays, 67601, Kansas, USA; 3 Fort Hays State University, Department of Biological Sciences, Hays, 67601, Kansas, USA; 4 Freie Universität Berlin, Institut für Biologie, Berlin, 14195, Germany

## Abstract

Depth and evenness of sequencing coverage are considered potential indicators of genome assembly quality. In plastid genomics, where new data generation has outpaced the development of assembly quality indicators, these coverage metrics could offer insights into the quality of plastomes of different sizes, structures, or taxonomic origins. However, the variation of sequencing depth and evenness among archived plastid genomes, their variability between genome partitions, and any association with methodological factors have yet to be evaluated. This study explores the variation of sequencing depth and evenness across a sample of publicly accessible plastid genomes in relation to their genome structure, assembly quality, and methodological provenance using uni- and multivariate statistical analyses. We also evaluate whether sequencing evenness in plastid genomes is biased by phylogenetic signal and assembly software choice, and whether more uniformly distributed input sequence data improves plastome assembly quality. Our results indicate significant differences in sequencing depth across the four structural partitions and between the coding and non-coding regions of plastid genomes, a significant correlation between sequencing evenness and the number of ambiguous nucleotides, and a significant difference in sequencing evenness between sequencing platforms. However, we also find that different covariates representing additional, lesser explored factors often show a similar, if not greater, explanatory power for the coverage variation. No indications of phylogenetic or software choice bias on sequencing evenness and only weak indications of phylogenetic bias among the assembly quality metrics are detected, suggesting that our study results represent genuine patterns. We also find that normalizing the distribution of the input sequence data before plastome assembly may improve assembly accuracy. Taken together, these findings highlight that many public plastid genomes derive from sequence data with highly variable depth and evenness, and that this variation is influenced, at least partially, by genome structure as well as methodological factors.

## Introduction

Publicly available plastid genomes are in need of standardized evaluations of assembly quality. More than 25,000 unique and complete plastid genome records have been submitted to NCBI Nucleotide as of May 2025, but a non-trivial proportion of them appear to exhibit manifestations of genome misassembly^1^. Multiple studies have reported widespread assembly and associated annotation errors among published plastid genomes, including incorrect gene or exon-intron boundaries^2^, missing or non-identical inverted repeats (IRs)^1,3,4^, and chimeric plastome-bacteria sequences^5^. Until improved assessment methods of plastome quality are developed, such errors will likely continue to accumulate.

Depth and evenness of sequencing coverage are considered possible indicators of genome assembly quality^6^. A relatively even sequencing coverage is seen as indicative of an unbiased genome assembly, as all genome regions are covered by a similar number of sequence reads^7^. By contrast, suboptimal genome assemblies often exhibit uneven coverage, as misassembled regions are poorly, if at all, covered^8^. Multiple studies have highlighted the importance of sequencing coverage when assessing the assembly quality: sequencing depth was employed to measure accuracy of *de novo* plastome assemblies^9,10^; sequencing evenness was used to identify or refine inconsistencies in plastome assemblies^11,12^; uneven sequencing coverage was discussed and evaluated for plastome assembly using long-read sequencing^13^; and plastome-wide sequencing depth was presented as a measure of coverage adequacy^14^. However, studies have also shown systematic coverage variation across different plastome regions, particularly those with uneven GC content^6,15^, possibly linked to coverage variation between coding and non-coding genome regions^16^. While sequencing depth and evenness may, thus, be effective indicators of plastome assembly quality, uncertainty about their associations with – or even bias by – various genome characteristics remains, limiting their applicability.

Several metrics to measure depth and evenness of sequencing coverage have been devised. Most of these metrics are agnostic to the size, structure, or taxonomic origin of the target genomes. Sequencing depth is typically measured as the number of times any nucleotide position of a genome is covered by sequence reads used to assemble it^7^; it is calculated as a genome-wide average, and multiple depth levels for reliable assembly or variant detection have been proposed for nuclear genomes^17^. Sequencing evenness is typically measured as the standard deviation around the mean of the normalized sequencing coverage^18^, and several studies have performed sensitivity analyses to determine meaningful evenness thresholds for nuclear genome sequencing^19^. Sequencing evenness can also be measured by a separate evenness score (‘E-score’)^20^, which represents the complement of the fraction of reads needing redistribution (from above-average to below-average coverage sections) to achieve even coverage; the E-score is positively correlated with the evenness of coverage depth but agnostic to its absolute value^18^. While each of these metrics can be easily calculated via computational implementations^19,21^, they remain under-utilized in plastid genomics^22^.

The utility of sequencing depth and evenness as indicators of plastome assembly quality should be evaluated together with measures of assembly quality. For example, depth and evenness should be assessed alongside plastid genome structure due to its potential link to assembly quality^14^. Most land plants exhibit a quadripartite plastid genome with a large (LSC) and a small single-copy (SSC) region, separated by two identical but reverse-complemented IRs (IR_A_, IR_B_)^23^. This genome structure is maintained during plastome replication^24^ and, thus, evolutionarily conserved^25^. Equality in IR length and sequence can, consequently, serve as a measure of assembly quality^22,26^, at least for the plastid genomes of most land plants^1^. Sequence contiguity and uncertainty represent additional measures of assembly quality that warrant comparison with sequencing coverage, especially among plastid genomes^27^. Contiguity (e.g., NA50) is the shortest contig length covering a percentage of the target sequence (e.g., 50%) and is common in nuclear genome assembly^28^. Uncertainty (e.g., the number of ambiguous nucleotides per sequence) is a direct measure of assembly quality^26^ but may be sensitive to organellar heteroplasmy and the choice of assembly software. Both of these assembly quality measures are expected to correlate with sequencing coverage. Comparing sequencing depth and evenness across structurally or taxonomically diverse plastid genomes would clarify the reliability of sequencing coverage as plastome quality metric.

Using sequencing depth and evenness to assess plastome assembly quality first requires testing the impact that different assembly software and sequencing platforms have on sequencing coverage. Various studies have reported that the choice of assembly software and next-generation sequencing (NGS) platform can affect sequencing coverage and, in turn, assembly quality^29^, including among plastid genomes^9^. For example, many *de novo* plastome assembly software tools fail to assemble complete plastomes^30,31^, instead generating multiple contigs that need post-processing and concatenation^32^. Scaffolding such contigs can introduce gaps and ambiguous nucleotides into the assembly^33,34^, biasing sequencing coverage^10^. Similarly, various sequencing platforms exhibit platform-specific error rates^35^ that can lead to non-trivial amounts of erroneous reads^36^. Since such errors are rarely random, platform choice may bias sequencing coverage^37^. The idiosyncrasies of different assembly software and sequencing platforms may, thus, confound sequencing coverage, warranting evaluation. Although testing all commonly used assembly software and sequencing platforms is beyond this investigation, a limited test can reveal whether assembly software or sequencing platform choice can significantly affect sequencing coverage.

In this investigation, we explore the variation of sequencing coverage across a sample of publicly available plastid genomes and test for associations between sequencing coverage and various plastid genome characteristics as well as methodological factors using statistical analyses. Specifically, we aim to evaluate if sequencing depth and evenness show differences across structural plastome partitions, correlations with IR equality or sequence uncertainty, and differences among select sequencing platforms and assembly software at a significant level. As corollaries, we also evaluate if sequencing evenness and measures of assembly quality exhibit phylogenetic bias, whether related factors such as partition and average read length similarly explain variation in sequencing depth and evenness, whether sequencing evenness in plastid genomes is biased by the choice of assembly software, and whether more uniformly distributed short-read sequence data can improve plastome assembly quality. For practical purposes, we also assess whether more uniformly distributed short-read sequence data has a measurable effect on plastome assembly quality.

## Methods

### Study design and hypotheses

To explore the variation of sequencing depth and evenness across publicly available plastid genomes, we retrieved a sample of more than 200 archived plastid genomes, their raw sequence reads, and their metadata from NCBI, measured the dispersion of sequencing depth and evenness across these genomes, and tested various hypotheses regarding that variation using uni- and multivariate statistical analyses. The tests on sequencing depth variation aimed to identify significant differences between, or associations with, factors related to plastid genome structure; the null hypotheses were: (i) sequencing depth variation is not significantly different between, or associated with, the four structural partitions (i.e., the ‘structural quadripartition’, comprising of the LSC, IR_B_, SSC, and IR_A_), and (ii) sequencing depth is not significantly different between, or associated with, the coding and the non-coding regions (i.e., the ‘coding/non-coding subdivision’) of plastid genomes. The tests on sequencing evenness variation aimed to identify correlations with plastome assembly quality and significant differences between, or associations with, sequencing platforms and assembly software; the null hypotheses were: (iii) sequencing evenness is not correlated with ambiguous nucleotides of plastome, (iv) sequencing evenness is not correlated with nucleotide mismatches between the two plastome IRs, (v) sequencing evenness is not significantly different between, or associated with, the platforms used for genome sequencing; and (vi) sequencing evenness is not significantly different between, or associated with, the software used for genome assembly. Several of these tests also included control variables to assess their potential confounding effects. Taken together, these tests aimed to identify relevant differences in or associations between sequencing coverage and factors related to genome structure, data quality, and methodological provenance.

### Compilation of plastome data set

To enable comparison with previous work, we evaluated the same plastid genomes as analyzed by Freudenthal et al.^9^, selecting records that met the following criteria: accessibility of the raw sequence reads via NCBI SRA^38^, a quadripartite genome structure, and a gene content that differs from the maximum gene set by no more than two annotations. The criterion of similar gene content ensured the validity of sequencing depth comparisons between coding and non-coding plastome sections. A total of 194 seed plant plastid genome records were thus selected, spanning various taxonomic lineages, sequencing platforms, and assembly methods. Accession numbers (NCBI Nucleotide and SRA) and taxonomic identities of these records are provided in Supplementary Table S1. Sequence data and metadata of each genome record were retrieved from NCBI: complete genome sequences were obtained from NCBI Nucleotide with Entrez Direct v.13.9^39^, corresponding sequence reads from NCBI SRA with SRA Toolkit v.2.10.8^40^. Assembly software names were parsed from NCBI Nucleotide, average read length and sequencing platform names from NCBI SRA; spelling errors were manually corrected. Extracted metadata values are listed in Supplementary Table S2. We replicated the quality filtering of the original plastome assemblies using a conservative approach: sequence quality and read pairing were assessed with Trimmomatic v.0.39^41^, retaining only paired reads >36 bp after removing all terminal nucleotides with a quality score <3. Since IR annotations of archived plastid genomes are frequently incorrect^1^, we evaluated and, where necessary, corrected IR start and end positions using script 4 of Gruenstaeudl et al.^42^, but did not assign new annotations where none existed.

### Calculation of sequencing depth and sequencing evenness

**Figure 1.**
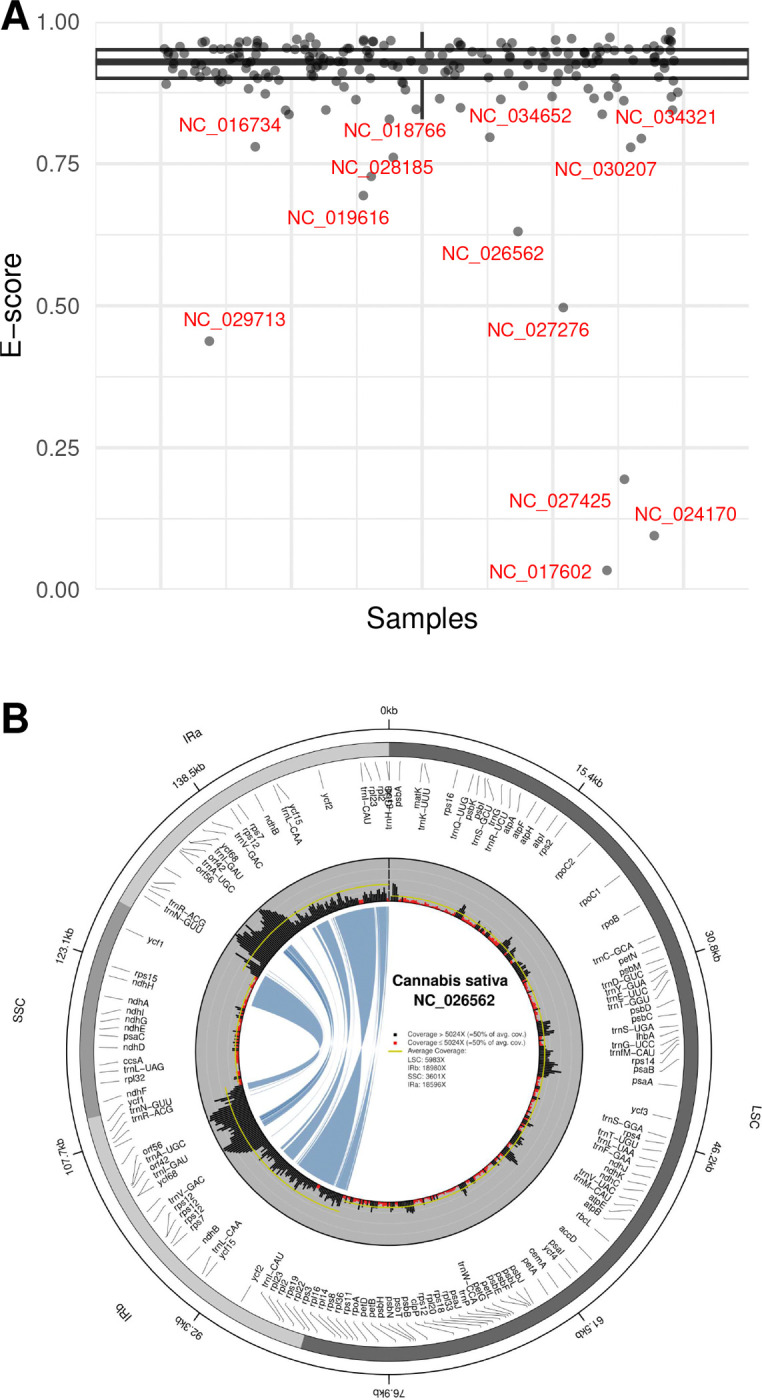
Dispersion of E-scores and an example of uneven sequencing coverage. “(**A**) Dispersion of E-scores for all analyzed plastid genomes. Outliers are highlighted in red and labeled with their NCBI Nucleotide accession numbers. (**B**) Sequencing coverage of the *Cannabis sativa* plastid genome (NC_026562), illustrating highly uneven coverage. With an E-score of 0.63, this plastome was classified as an outlier in the E-score distribution. Red bars in the circular histogram mark windows with sequencing depth less than half the genome-wide average.

Sequencing depth and evenness were calculated for each plastid genome under study. Sequencing depth was calculated with PACVr v.1.1.3^22^ after mapping quality-filtered reads to each genome using Bowtie2 v.2.4.2^43^ and indexing with Samtools v.1.9^44^. To avoid double-counting reads at the IRs, indexing settings allowed each read only one best-position mapping. All depth calculations used 250 bp windows, with the average plastome-wide depth inferred across all windows. Coverage windows with sequencing depth at least one standard deviation (s) below the plastome-wide average were classified as ‘windows with reduced sequencing depth’ (WRSD) and categorized by structural quadripartition and coding/non-coding subdivision. WRSD outlier values beyond Tukey’s ‘far out’ fences of ±3 interquartile range (IQR)^45^ were excluded before final analysis. Sequencing evenness was calculated as the plastome-wide E-score of Oexle^18^ using PACVr. E-scores range from 0 to 1, with values near 1 indicating even and values near 0 indicating uneven coverage. Preliminary analyses indicated that E-scores ≤ 0.80 represented highly uneven sequencing coverage (Fig. 1). Thus, we flagged all E-scores that were more than 3×IQR below the first quartile of the dataset-wide E-score distribution (i.e., Tukey’s ‘far out’ lower fence) as outliers and excluded them before final analysis. The WRSD count by genome partition and coding status, and the E-score for each plastid genome, are listed in Supplementary Table S2.

### Calculation of assembly quality metrics

For each plastid genome, we calculated two assembly quality metrics: the number of ambiguous nucleotides and the number of nucleotide mismatches between its IRs. Both metrics are established indicators of assembly quality and completeness^46,47^, including for plastid genomes^26,48^, and are negatively correlated with assembly quality (i.e., lower values indicate higher accuracy and, thus, higher assembly quality). We calculated both metrics using R scripts^49^ from PACVr. Prior to IR mismatch inference, we verified gene content and order across IRs and excluded plastomes lacking IR synteny. After calculating the metrics, we standardized the values using Tukey’s ladder of powers^50^, a statistically neutral transformation that improved comparability; the resulting values ranged from 0 to 1, with values near 1 indicating high assembly quality (i.e., few ambiguous nucleotides or IR mismatches) and those near 0 low quality. Untransformed assembly quality values are listed in Supplementary Table S2.

### Classification of variables

To support the statistical analyses, we classified all variables under study as quantitative or categorical: the quantitative variables included the plastome-wide E-score, WRSD counts per genome partition or coding status, ambiguous nucleotides per genome, IR sequence mismatches, partition length, and average read length, the categorical variables the type of sequencing platform and the assembly software used. To ensure balanced test statistics across the categorical variables^51^, we set a minimum of five genomes per state; states below this were removed, and their members classified as ‘missing’. To account for genome length differences in the statistical analyses, we applied two standardizations: WRSD counts were normalized by partition length to account for length differences between the structural partitions, and by gene and intergenic spacer length to account for length differences between coding and non-coding plastome sections; specifically, each WRSD count was divided by cumulative partition or section length and expressed per 1000 bp. Homoscedasticity was tested in all variables using Levene’s test^52^. As homoscedasticity was confirmed only for variables related to sequencing evenness, we used only non-parametric univariate tests to evaluate sequencing depth.

### Statistical testing of hypotheses

Both uni- and multivariate statistical analyses were applied to test the hypotheses of this investigation. Univariate analyses were used to assess whether variation in sequencing depth or evenness was significantly associated with individual categories of the genome structure or methodological provenance variables – for example, whether different structural or coding partitions differed significantly in their depth variation, or whether evenness differed significantly with sequencing platform or assembly software choice. In comparison, multivariate analyses were used to assess which of multiple variables were associated with variation in sequencing depth or evenness when analyzed in concert, and whether the inclusion of control variables changed these results, indicating confounding. For example, multivariate analyses were used to test whether structural quadripartition, the coding/non-coding subdivision of plastid genomes, or both were significantly associated with sequencing depth variation, and whether control variables had similar or greater explanatory power. Similarly, multivariate analyses were used to test whether the number of ambiguous nucleotides, IR mismatches, or the choice of sequencing platform — either isolated or in combination – was significantly associated with sequencing evenness, and whether control variables had a similar, if not higher, explanatory power. In addition, multivariate analyses were used to examine estimations of sequencing depth or evenness values across specific predictor and covariate levels, leveraging the iterative data partitioning by regression trees to model the mean outcome within each subset.

### Univariate statistical tests

The hypotheses that sequencing depth does not differ significantly across structural quadripartition (hypothesis i) and that sequencing evenness does not differ significantly across sequencing platforms (hypothesis v) or assembly software (hypothesis vi) were tested using non-parametric ANOVA to detect differences among ≥ 3 independent groups. Specifically, we used Kruskal-Wallis^53^ and post-hoc pairwise Wilcoxon rank-sum tests^54^ to assess whether WRSD counts differed significantly among plastome partitions and whether E-scores differed significantly across sequencing platforms or assembly software. Benjamini-Hochberg correction^55^ was applied to account for multiple testing^56^. The hypothesis that sequencing depth does not differ significantly between coding and non-coding plastome sections (hypothesis ii) was tested non-parametrically with a Wilcoxon rank-sum test^57^, assessing whether WRSD counts differed significantly between these categories. Since the data contained ties, we calculated the test statistic using normal approximation. The hypotheses that sequencing evenness is not correlated with the number of ambiguous nucleotides (hypothesis iii) or IR mismatches (hypothesis iv) per plastome were tested parametrically using Spearman’s rank statistic^58^, assessing whether the E-score was significantly correlated with either assembly quality metric. The correlation coefficient *R*_*s*_ was considered small if *R*_*s*_ ≥ 0.1, moderate if *R*_*s*_ ≥ 0.3, and large if *R*_*s*_ ≥ 0.5. The relevance of the p-values of the Kruskal-Wallis and Wilcoxon rank-sum tests^59^ was evaluated through effect sizes using the *η*^2^ H-statistic^60^ and Cohens-d (*d*_*c*_)^61^, respectively. P-values below *α* = 0.05 were considered significant, below *α* = 0.01 very significant, and below *α* = 0.001 highly significant. Effect sizes were considered small if *η*^2^ ≥ 0.01 or *d*_*c*_ ≥ 0.2, moderate if *η*^2^ ≥ 0.06 or *d*_*c*_ ≥ 0.5, and large if *η*^2^ ≥ 0.14 or *d*_*c*_ ≥ 0.8.

### Multivariate statistical tests

Five of the study hypotheses were also tested with one parametric and one non-parametric method of multivariate statistical analysis: linear regression and regression trees. For both methods, we specied regression models with multiple predictors, one outcome, and optionally two control variables (covariates). Associations between sequencing depth and structural quadripartition (hypothesis i) and coding/non-coding subdivision (hypothesis ii) of the plastomes were tested by modeling each variable as a separate predictor and treating each partition as independent category of that outcome. Analogously, associations between sequencing evenness and ambiguous nucleotides (hypothesis iii), IR mismatches (hypothesis iv), and sequencing platform choice (hypothesis v) were tested by modeling each variable as a predictor and treating each platform as an independent category. Assembly software choice (hypothesis vi), by contrast, was excluded from the multivariate analyses to avoid biased results, as this variable contains a high proportion of missing data, making the ‘missing’ category the most prevalent and potentially influential. To assess the potential for confounding, each multivariate analysis was conducted with and without the inclusion of two covariates: total partition length and average read length. These variables may be the underlying drivers of associations with structural quadripartition and sequencing platform choice, respectively, and were thus included in some analyses. All regression models were implemented in R using the package tidymodels^62^. For linear regression, the adjusted R-squared value was used as a metric to estimate the proportion of outcome variance explained by the predictors^63^. For regression trees, the root mean squared error (RMSE) was used as the loss function^64^.

### Tests for phylogenetic independence

Sequencing depth is largely determined by methodological factors – such as genomic library preparation, average read length, and choice of sequencing platform – and is, thus, generally unaffected by plastid genome evolution^65,66^. In contrast, sequencing evenness can be shaped by inherited sequence features like GC content and repetitive elements^67,68^ and may, consequently, mirror the evolutionary history of the underlying genomes: closely related plastid genomes may share similar sequencing evenness due to inherited sequence-dependent traits^69,70^. Any investigation on sequencing evenness should, therefore, evaluate if the variables under study are phylogenetically biased. To evaluate whether statistical differences in, or associations with, sequencing evenness in this study reflect true genetic patterns rather than phylogenetic similarity, we conducted tests for phylogenetic independence. The concept of phylogenetic independence denotes the absence of trait similarity attributable to shared evolutionary history, in contrast to phylogenetic signal, where closely related species exhibit similar traits^71^. To avoid phylogenetic bias in our analyses, we evaluated whether the E-score and its two hypothesized quantitative predictors – the number of ambiguous nucleotides and the number of IR mismatches per plastid genome – exhibited phylogenetic signal. The tests were conducted on a phylogenetic tree representing the evolutionary relationships among all 194 plastid genomes under study (Supplementary Fig. S1). The tree was inferred under maximum likelihood (ML) from a sequence alignment of all the genes shared across the input plastomes. Specifically, the same 86 genes were extracted from each plastome and aligned gene-wise based on translated amino acid sequences using the Python package plastomeburstalign^72^. The best ML tree was then inferred from the concatenated alignments under a GTR nucleotide substitution model using raxml-ng^73^. For each of the three evenness measures potentially biased by phylogenetic similarity, three statistical indices of phylogenetic signal were calculated: *I*^74^, which calculates phylogenetic signal based on trait values and patristic distances along a tree using spatial autocorrelation; *K*^75^, which compares observed trait variance on a tree to expectations under Brownian motion; and K*^75^, which is a standardized version of *K* that enables comparisons of phylogenetic signal across trees of different size and topology. Since these indices are sensitive to factors such as the proportion of missing values and the number of trait classes^76^, we computed each index for three separate trait distributions to contextualize the results: the empirical distribution, a randomized distribution, and a distribution with enforced phylogenetic signal. The randomized distribution retained the mean, dispersion, and proportion of missing values of the empirical distribution and lacked phylogenetic signal, whereas the distribution with enforced phylogenetic signal was simulated along the best ML tree under a Brownian motion model. This setup allowed us to compare the empirically derived index values to those obtained from controlled trait distributions: one lacking phylogenetic signal and one with strong phylogenetic signal. Each index of phylogenetic signal was computed both as a global, tree-wide value and as an autocorrelation function across the best ML tree, enabling a detailed visualization of phylogenetic signal relative to phylogenetic distance^77^. Signal-distance relationships were visualized using phylogenetic autocorrelograms^74^, with 95% confidence intervals estimated via non-parametric bootstrap resampling. All index calculations and correlogram visualizations were performed in R using the R package phylosignal^77^.

### Evaluation of evenness bias from assembler choice

Many of the 194 plastid genomes analyzed for sequence evenness in this study were assembled using different plastome assembly software. Consequently, observed differences in evenness may reflect assembler choice rather than inherent genomic characteristics. This possibility is supported by Freudenthal et al.^9^ who reported that different assembly tools can generate substantially divergent plastid genome sequences from the same sequence input data. To test whether assembly software choice can bias sequencing evenness in plastid genomes, we compared E-scores across plastome assemblies generated from identical short-read sequence data but different assembly software, using some of the assemblies generated by Freudenthal et al.^9^ as reference data. However, many of their plastome assembly products consisted of multiple small contigs rather than complete circular plastome sequences, which would prevent accurate E-score calculation. Therefore, we selected only those samples of Freudenthal et al.^9^ where at least four plastome assemblers produced complete or near-complete plastid genomes (i.e., single best contig ≥ 100,000 bp). This criterion was met by 20 samples given the use of the plastome assemblers GetOrganelle^32^, fast-plast^78^, ORG.asm^79^, and NOVOPlasty^30^. For each sample, we mapped the corresponding short-read sequences onto the best contig per assembler using Bowtie2 and calculated the E-score with PACVr.

### Normalized sequence reads and assembly quality

Unevenly distributed short-read sequence data is known to result in suboptimal genome assemblies^8,68^. To assess whether more uniformly distributed sequence data leads to plastome assemblies of higher quality, we performed *de novo* plastome assembly on normalized short-read sequence data of multiple samples and evaluated each resulting assembly using the two assembly quality measures (i.e., the number of ambiguous nucleotides and the number of IR mismatches per plastome). To obtain relatively uniformly distributed short-read sequence data for each sample, we first capped sequencing depth at 100X using jvarkit^80^ and then applied a Kmer-based read normalization to the capped sequence data using BBNorm^81^. Successful read normalization was confirmed with PACVr (Supplementary Fig. S2). *De novo* plastome assembly was conducted on the normalized short-read data using GetOrganelle, and the assembly quality measures were calculated using PACVr.

## Results

### Extracted genome metadata

Sequencing platform and assembly software names were extracted for most of the input genomes, but only a subset met our criteria for statistical analysis (Table 1). Sequencing platform names were extracted for all but two plastid genomes and grouped into 12 states; of these, four states failed the sample size requirement and eight samples were excluded as outliers, leaving eight final states with 178 genomes (excluding the state ‘missing’) . Among these, the Illumina platforms HiSeq 2000, HiSeq 2500, and MiSeq were the most frequent. Assembly software names were extracted for 73 plastid genomes and grouped into 15 states; of these, eleven states failed the sample size requirement and three samples were removed as outliers, leaving four final states with 45 genomes (excluding the state ‘missing’). Among these, the software Velvet^82^ and CLC Genomics Workbench^83^ were the most frequent. Although our strict sample size and outlier criteria excluded many common sequencing platforms and assembly tools from the data set, the final character states met rigorous standards for statistical analysis, enabling reliable tests of statistical difference and association.

### Variability in sequencing depth

**Figure 2.**
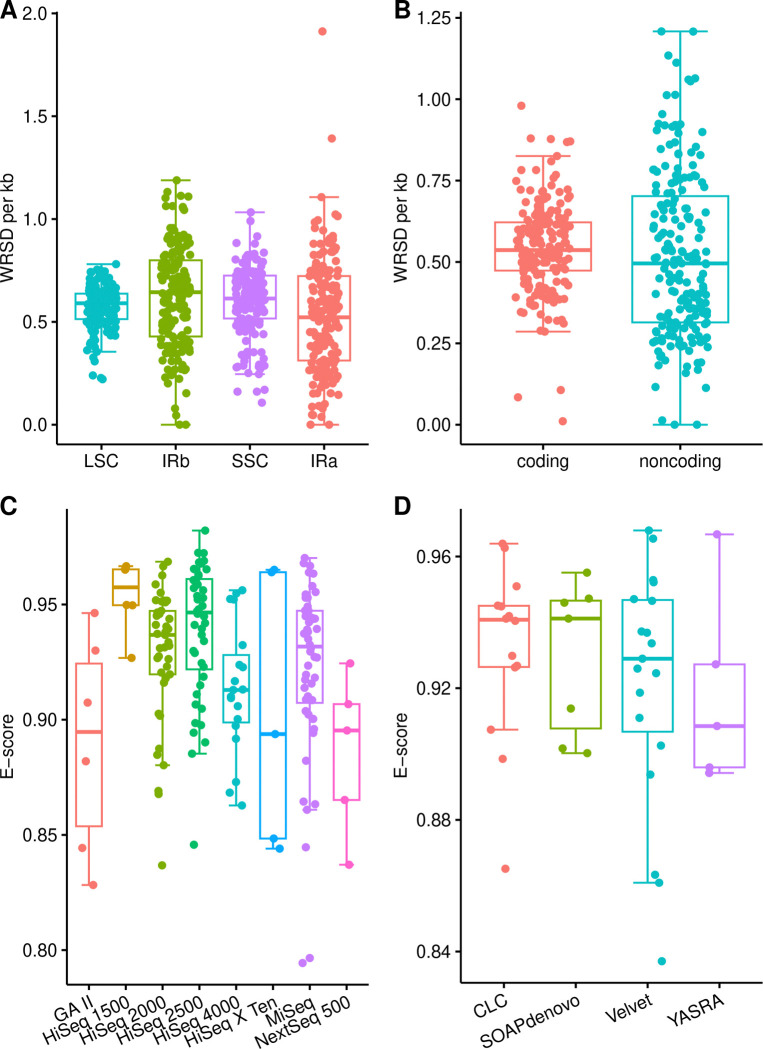
Dispersion of WRSD counts and E-scores by structural genomic and methodological factors. Box plots show WRSD count dispersion by (**A**) structural quadripartition and (**B**) coding/non-coding subdivision, and E-score dispersion by (**C**) sequencing platform and (**D**) assembly software. Variable states are color-coded for clarity.

Considerable variability in WRSD counts was observed across the structural quadripartition and the coding/non-coding subdivision of the plastid genomes (Table 2). WRSDs were detected in all four structural partitions in 165 (85.1%) of the plastid genomes. After outlier removal, the mean WRSD count was 0.57 for the LSC, 0.63 for the IR_B_, 0.60 for the SSC, and 0.53 for the IR_A_. In all partitions except IR_A_, the median WRSD count exceeded the mean, indicating a negative data skew (i.e., an overrepresentation of large WRSD values). WRSD dispersion in the IRs was found to be slightly wider than in the single-copy regions, especially relative to the LSC (Fig. 2A). WRSDs were identified among both coding and non-coding regions in all plastid genomes. After outlier removal, the mean WRSD count was 0.55 for coding and 0.52 for non-coding regions. Comparing the median and mean WRSD counts across all coding and non-coding regions indicated a positive data skew (i.e., an underrepresentation of large WRSD values). WRSD dispersion in the non-coding regions was found to be considerably wider than in the coding regions (Fig. 2B).

### Variability in sequencing evenness

Considerable variability in E-scores among the plastid genomes was observed, with some identified as outliers in the overall distribution. The plastome-wide E-score averaged 0.90, with an interquartile range (IQR) of 0.90—0.95 across all plastid genomes (Table 2). The median E-score exceeded the mean, indicating a negative data skew (i.e., an overrepresentation of large E-scores). The highest E-score was observed in the plastid genome of *Asparagus officinalis* (NC_034777; E-score = 0.98), the lowest in that of *Elaeis guineensis* (NC_017602; E-score = 0.03); the latter plastome was among 13 plastid genomes identified as E-score outliers (Fig. 1A; labeled in red), all with E-scores ≤ 0.80, indicating a highly uneven sequencing coverage. The coverage profiles of these outliers are visualized in Fig. 1B and Supplementary Figs. S3–S15.

### Variability in assembly quality metrics

Considerable variability in the two assembly quality metrics was observed across the plastid genomes (Table 2). The number of ambiguous nucleotides per plastome ranged from 0 to 551, with the highest value found in *Stachys byzantina* (NC_029825; Supplementary Table S2). The number of IR sequence mismatches per plastome ranged from 0 to 12,830, with the highest value found in *Dendrobium nobile* (NC_029456); the latter values is unusually high and resulted from an assembly error that arranged the IRs as tandem repeats rather than reverse complements. In all other plastid genomes analyzed, IR mismatches did not exceed 284 nucleotides (Supplementary Table S2).

### Univariate tests on sequencing depth

The univariate tests on sequencing depth revealed significant differences in WRSD count distributions across the four structural partitions and between coding and non-coding plastid regions. The Kruskal-Wallis test detected significant differences in WRSD count distribution across the four structural partitions (*p* < 0.001; Table 3), although with a small effect size (*η*^2^ = 0.030). Post-hoc Wilcoxon rank-sum tests also found significant differences between most of these partitions (except between IR_B_ and the SSC) and, similarly, between the coding and non-coding plastome regions, again with a small effect sizes (Table 4). Based on these results, we reject hypotheses (i) and (ii) and conclude that WRSD counts and, by extension, sequencing depth varies significantly by structural quadripartitions as well as by coding/non-coding subdivision – at least among the plastid genomes analyzed.

### Univariate tests on sequencing evenness

**Figure 3.**
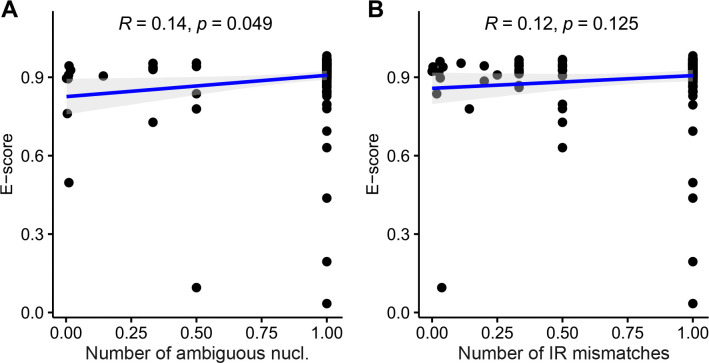
Correlation plots showing the relationship between the E-score and each of two assembly quality metrics. The plots show the correlation between the plastome-wide E-score and (**A**) the number of ambiguous nucleotides and (**B**) the number of IR nucleotide mismatches per plastome. Both quality metrics were scaled to a range of 0 to 1, where lower values indicate more ambiguous nucleotides or IR mismatches, respectively. Regression lines are displayed in blue, their 95% confidence intervals in light gray.

The univariate tests on sequencing evenness revealed a significant correlation between E-scores and the number of ambiguous nucleotides, but not with the number of IR mismatches. Spearman rank correlation tests detected positive correlations between plastome-wide E-scores and both assembly quality metrics, but only the correlation with the ambiguous nucleotide count was significant (*R*_*s*_ = 0.14, *p* = 0.049; Fig. 3), the correlation with the IR mismatch count was not (*R*_*s*_ = 0.12, *p* = 0.125). Based on these results, we reject hypotheses (iii) but not hypothesis (iv) and conclude that the plastome-wide E-score and, by extension, sequencing evenness is correlated with the number of ambiguous nucleotides per plastome – at least among the plastid genomes analyzed. The univariate tests of sequencing evenness also revealed significant differences in E-scores across sequencing platforms but not across assembly software tools. The Kruskal-Wallis test revealed significant differences in plastome-wide E-scores across sequencing platforms (*p* < 0.001; Table 3) with a moderate effect size (*η*^2^ = 0.120). Similarly, post-hoc Wilcoxon rank-sum tests identified seven (25%) of 28 possible comparisons as significant, with six exhibiting a large effect size (Supplementary Table S3). A comparison of the E-score distributions across sequencing platforms indicated that HiSeq 1500 had a narrower dispersion than most other platforms (Fig. 2C). In contrast, plastome-wide E-scores did not differ significantly across assembly software tools (*p* = 0.729; Table 3), even though SOAPdenovo showed a slightly narrower E-score distribution than the other software tools evaluated (Fig. 2D). Based on these results, we reject hypotheses (v) but not hypothesis (vi) and conclude that the plastome-wide E-score and, by extension, sequencing evenness varies significantly between sequencing platforms – at least among the plastid genomes analyzed.

### Linear regression analyses

The multivariate linear regression models explained about one quarter of the variation in sequencing depth and evenness, with the remaining variation unexplained, indicating a moderate explanatory power of the model predictors. For sequencing depth, both predictors were found to be significant in explaining depth variation, especially the LSC and the SSC in structural quadripartition (Supplementary Tables S4). For sequencing evenness, each of the three predictors was found to be significant in explaining evenness variation, but only in connection with sequencing platform HiSeq 2000 (Supplementary Tables S5). Notably, the proportion of variance explained by the predictors was higher when covariates were excluded from the analyses, suggesting potential confounding: without covariates, the models explained 28.3% of the variation in sequencing depth and 26.8% in sequencing evenness, whereas with covariates included, the explained variation declined to 23.9% and 23.7%, respectively (Supplementary Tables S4 and S5).

### Regression tree analyses

**Figure 4.**
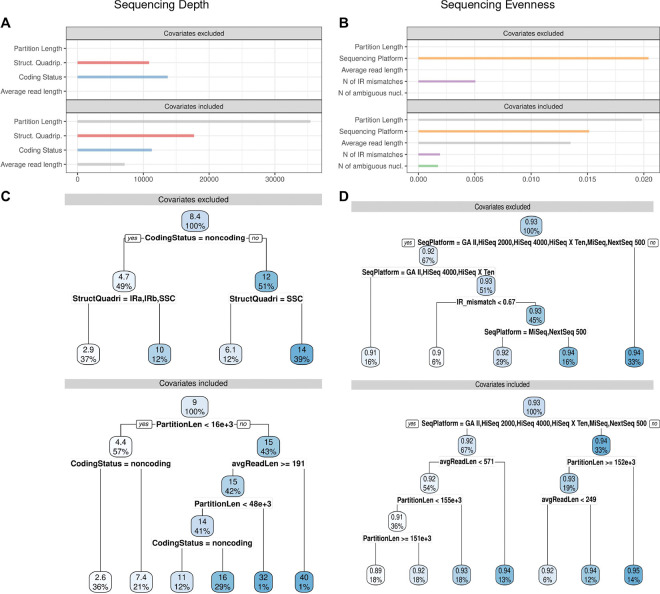
Variable importance and optimal data partitioning in regression tree models on sequencing depth and evenness. The variable importance plots show the relative importance scores of the predictors for (**A**) sequencing depth and (**B**) sequencing evenness in the best-fitting regression tree models, estimated with and without covariates. The regression trees illustrate the optimal data partitioning under the best-fitting models with and without the inclusion of covariates for (**C**) sequencing depth and (**D**) sequencing evenness.

The multivariate regression tree models showed similar explanatory power as the corresponding linear models, including a comparable decrease in explained variation when covariates were included: without covariates, the models explained 31.2% of the variation in sequencing depth and 25.9% in sequencing evenness; with covariates, the explained variation declined to 22.9% and 12.6%, respectively (Supplementary Tables S6 and S7). Including covariates in the regression tree models also led to changes in the variable importance scores. In the models for sequencing depth, structural quadripartition and coding/non-coding subdivision initially showed moderate variable importance, whereas partition length became the most influential variable when covariates were included (Fig. 4A). For sequencing evenness, platform choice had a much higher variable importance than IR mismatch count, but partition length again emerged as most influential when covariates were included (Fig. 4B). Additionally, the covariate average read length had a substantially higher importance score for explaining evenness variation than either assembly quality metric; in contrast, the number of ambiguous nucleotides explained evenness variation minimally, regardless of covariate inclusion (Fig. 4B). The structure of the best-fitting regression trees reflected the relative importance of different model predictors and covariates. In the regression tree for sequencing depth, the top split was the coding/non-coding subdivision under the exclusion of covariates, but shifted to partition length when covariates were included, highlighting the importance of this control variable in explaining sequencing depth variation (Fig. 4C). Without covariates, the SSC and IR regions were more often linked to lower sequencing depth than the LSC, whereas with covariates, non-coding regions were more frequently associated with lower depth than coding regions (Fig. 4C). In the regression tree for sequencing evenness, sequencing platform consistently formed the top split, highlighting its dominant role in explaining evenness variation. With covariates included, partition length and average read length appeared in all lower splits, underscoring their importance in the regression model (Fig. 4D). Notably, sequencing platforms such as HiSeq 2000, HiSeq 4000, and HiSeq X Ten were repeatedly linked to lower evenness at top-level splits, regardless of covariate inclusion, suggesting an association between specific platforms and reduced evenness.

### Tests for phylogenetic independence

**Figure 5.**
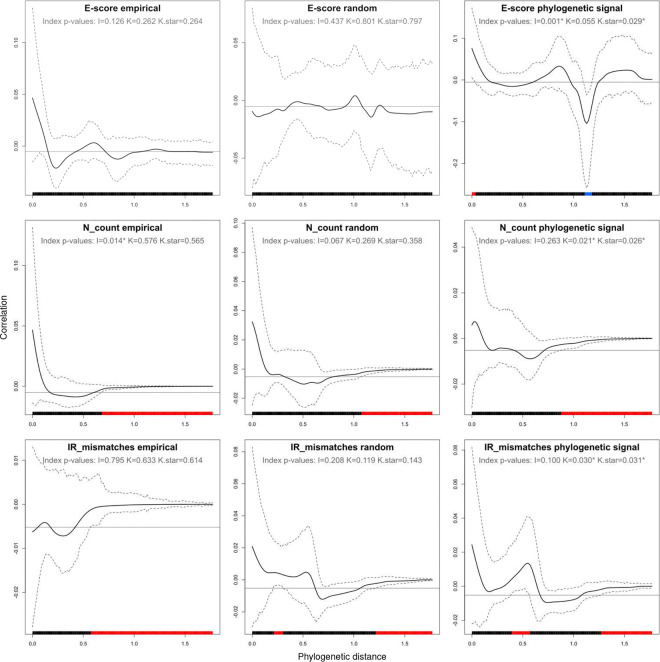
Phylogenetic autocorrelograms for three metrics related to sequencing evenness based on empirical and test trait distributions. The correlograms represent the E-score (top row), the number of ambiguous nucleotides per plastome (middle row), and the number of IR mismatches per plastome (bottom row) under three trait distributions: empirical (left column), randomized (middle column), and with enforced phylogenetic signal (right column). The black curve represents the autocorrelation index across phylogenetic distances, with dashed lines indicating its 95% confidence interval. The horizontal line marks the expected index value under the null hypothesis. The colored x-axis highlights phylogenetic distances with significant autocorrelation (red for positive, blue for negative autocorrelation).

When the E-score and the two assembly quality metrics are mapped onto the best ML tree, no clear phylogenetic pattern is evident: although the tree topology reflects the known evolutionary relationships among seed plants^84^ – with each analyzed plant order and most major angiosperm clades (e.g., magnoliids, monocots, asterids, rosids) recovered as monophyletic – the variation in these evenness measures does not appear to be clade-specific (Supplementary Fig. S1). However, the number of ambiguous nucleotides and the number of IR mismatches per plastome were found to vary widely within a broad range, while the E-score showed lower average dispersion, indicating its greater suitability as a statistical indicator for multivariate analyses^85^. The phylogenetic autocorrelograms showed no evidence of phylogenetic bias for the E-score: the empirical E-score distribution closely resembled the random E-score distribution, both aligning with the expectation of the null hypothesis, whereas the E-score distribution with enforced phylogenetic signal deviated significantly from the null expectation near the Cartesian origin and a phylogenetic distance of 1.1 (Fig. 5). Similarly, the global phylogenetic signal indices *I* and K* were significant only under the enforced signal distribution, not under the empirical or random distributions. In contrast, autocorrelograms for the two assembly quality measures were harder to interpret, as neither the randomized nor the enforced distributions showed expected significance patterns: all three deviated from the null expectation at phylogenetic distances ≥ 0.6, making these distances unsuitable for signal detection. Below that distance threshold, expected patterns also failed to emerge: phylogenetic signal was absent in the ambiguous nucleotide count under both controlled trait distributions and present in the IR mismatch count under both. Therefore, we focused on the global phylogenetic signal indices, which exhibited consistent significance patterns: the empirical autocorrelogram for ambiguous nucleotide count deviated significantly from the null expectation only under one of the three indices, while the autocorrelogram for IR mismatch count showed no significant deviation. Thus, we concluded that neither the E-score nor the assembly quality metrics exhibit relevant phylogenetic signal in this study.

### Evaluation of evenness bias from assembler choice

**Figure 6.**
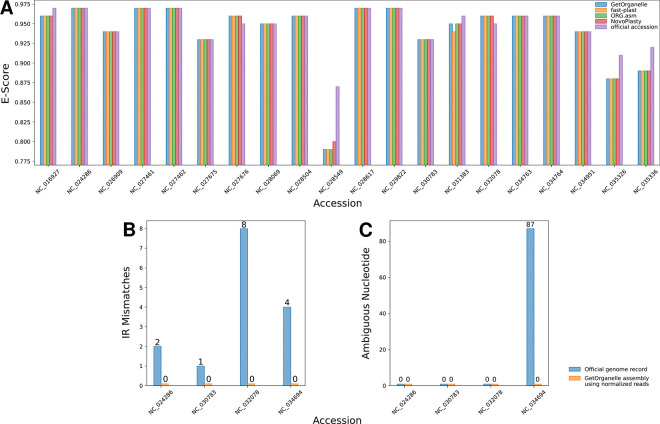
Assessment of sequencing evenness and plastome assembly quality across different assemblers and read normalization. (**A**) E-score comparison for plastome assemblies from four different assembly software tools and the official plastome sequence across 20 samples. Each assembly software is represented by a distinct color. Comparison of IR mismatches (**B**) and ambiguous nucleotide count (**C**) for plastome assemblies of identical samples before and after read normalization.

Our evaluation of whether sequencing evenness in plastid genomes is biased by assembly software choice showed that sequencing evenness variability was much lower across assembly software than across samples: in 18 of the 20 samples, all four plastome assemblers produced contigs with nearly identical E-scores (Fig. 6A). Only the official plastome sequence per sample showed different E-scores in seven of the samples, probably due to manual adjustments made by the genome authors after automated assembly. By comparison, the E-scores varied strongly between samples, ranging between 0.79 and 0.97. These results indicate that any differences in sequencing evenness identified in this study are unlikely due to bias from assembly software choice.

### Normalized sequence reads and assembly quality

Evaluating the effect of more uniformly distributed short-read sequence data showed that plastome assemblies from more evenly distributed reads exhibited higher quality than those from more uneven data. Complete, circular plastid genomes were successfully assembled by GetOrganelle both before and after read normalization from four samples. The number of ambiguous nucleotides and the number of IR mismatches showed considerable differences between these paired assemblies: the plastomes assembled from unevenly distributed sequence data consistently showed IR mismatches and, in one case, numerous ambiguous nucleotides, whereas the plastomes assembled from normalized sequence data showed neither (Fig. 6B,C). These results suggest that sequence read normalization may be an effective way to improve plastome assembly quality.

## Discussion

The results of this study indicate that sequencing depth differences across the structural quadripartition of plastomes are unlikely due to chance: WRSD count distributions differed significantly among all four partitions (except between the IR_B_ and the SSC; Table 4). These differences are not due to natural length variation among the partitions (i.e., the LSC is typically longer than the other partitions), as the WRSD data was length-standardized before univariate analysis. Even the two IRs, which are – by definition – identical in length, showed significant depth differences, albeit with small effect size. The IRs of a plastome are homogenized in length and sequence by recombination-dependent replication and natural gene conversion^24,86^; their WRSD counts should, thus, be nearly identical, yet significant differences were detected. Structural quadripartition was also found to be a strong predictor for sequencing depth in the linear regression analyses, with the LSC and the SSC significantly associated with depth variation (Supplementary Tables S4). Similarly, the regression trees indicated that the SSC and the IR regions were more frequently linked to lower sequencing depth than the LSC when covariates were excluded (Fig. 4C). However, partition length, which was defined as a covariate, was found to impact sequencing depth considerably, explaining more depth variation than structural quadripartition (Fig. 4A). Thus, from a statistical standpoint, structural quadripartition should be interpreted cautiously for explaining sequencing depth variation, as the variation may stem from additional, underexplored factors, such as uneven GC content, local genome duplications, or methodological bias. GC variation within a plastome has previously been suggested as a source of sequencing depth bias. For instance, heterogeneous GC content in Illumina data was reported to distort sequencing depth measurements^15,36,87^. Similarly, both GC-rich and GC-poor sequences reportedly caused an underestimation of local sequencing coverage^88^. Additionally, sequencing depth variation may result from integration of duplicated plastome regions into the nuclear or mitochondrial genome. In *Eucalyptus grandis*, duplicated plastid regions integrated into the mitochondrial genome showed 10-fold increases in sequencing depth^89^. A similar sequencing depth variation was reported for intracellular gene transfers in *Cynomorium*^90^. The sequencing depth differences observed here may also reflect GC bias or intracellular gene transfers, warranting further study. For the IR regions, methodological artifacts may be another explanation for sequencing depth differences. Genomic repeat regions are known to affect the bioinformatic read mapping to a reference genome^68,91,92^. Bowtie2, for example, uses pseudo-random numbers to break ties between identical mapping scores, which are common among repeats^43^; at low sequencing depths, this can lead to uneven coverage in these regions^93^. Moreover, repeat region borders challenge mapping software, as reads span junctions to non-repeat areas; plastome IRs may, thus, show different WRSD counts despite identical sequences^22^. To our knowledge, only one other study has evaluated sequencing depth across the structural quadripartition of plastid genomes, and they reported similar results^94^. However, the findings of this study should not be generalized to partition-specific sequencing depth differences without testing potential correlates such as GC content, intracellular gene transfer, or methodological bias.

The results of this study also indicate that sequencing depth differences across the coding/non-coding subdivision of a plastid genome are unlikely due to chance: WRSD count differed significantly between coding and non-coding plastome sections, although effect size was small (Table 4). Both mean and median WRSD counts in coding regions exceeded those in non-coding regions (Table 2); this difference cannot be attributed to coding regions’ greater cumulative length (for the genomes analyzed, the coding/non-coding length ratio is 2.7), as the WRSD data were length-standardized before univariate analysis. Instead, the sequencing depth differences, like those across the structural quadripartition, likely reflect understudied factors such as divergent GC content. Coding status was also strongly associated with sequencing depth variation in the linear regression analyses (Supplementary Table S4), and the regression trees identified it as a top split variable regardless of covariate inclusion (Fig. 4C). However, the covariate partition length was found to exhibit higher variable importance and explained more sequencing depth variation than coding status in the regression tree models (Fig. 4A), indicating that additional factors such as uneven GC content may influence the variation in sequencing depth. In nuclear genomes, GC content is typically higher in coding than non-coding regions, partly due to GC-biased gene conversion, a meiotic repair process favoring GC-rich alleles^95^. Similar patterns of gene-linked GC richness have been observed in many plastid genomes^96,97^, especially in genes in the IRs^98,99^. Future research should assess how divergent GC content affects sequencing depth differences between coding and non-coding plastome regions.

The results of this study also indicate that at least one of the observed correlations between sequencing evenness and assembly quality may be non-random: a significant correlation was found between the E-score and the number of ambiguous nucleotides (Fig. 3A). This finding supports the idea that poorly assembled genome regions exhibit uneven sequencing coverage^8^ and suggests using sequencing evenness as indicator to improve plastid assembly inconsistencies^11,12^. Moreover, we also found that plastome assemblies from more uniformly distributed short-read sequencing data exhibited higher genome quality than those from uneven sequence data: the latter consistently produced IR mismatches and, in one case, numerous ambiguous nucleotides – issues absent in the more uniformly distributed data (Fig. 6B,C). However, as with sequencing depth, correlation with evenness may be biased by additional, underexplored factors. For example, the regression tree analyses showed that average read length was a stronger predictor for sequencing evenness than either assembly quality metric (Fig. 4A). Several other confounding effects are possible as well. For instance, a correlation between sequencing evenness and the number of ambiguous nucleotides may result from mutations in duplicated plastome regions transferred to the nuclear or mitochondrial genome. Conventional assembly software cannot distinguish original plastid DNA from nuclear or mitochondrial paralogs and uses both for assembly and depth calculation. Hence, sequencing evenness may be low in plastid-to-nuclear or plastid-to-mitochondrial transfer regions, especially after whole-genome sequencing. Such effects on sequencing coverage are documented for nuclear mitochondrial DNA segments^100^, but remain largely unexplored for plastid transfers. In contrast, we found no indications that either the E-score or the two assembly quality measures exhibited relevant phylogenetic bias in our analyses (Fig. 5). Thus, all study results regarding sequencing evenness, if significant, likely reflect genuine patterns. The observation that both assembly quality metrics correlated positively with sequencing evenness, but only one significantly, may reflect their differing nature. Mismatches between IRs often stem from annotation errors, not true sequence differences^1^, and may not affect sequencing depth. In contrast, ambiguous nucleotide counts can arise from natural (e.g., heteroplasmy) or artificial (e.g., base call settings of assembly software) causes and typically affect depth calculation, making this measure sensitive to changes in sequencing evenness^30,101^. The extent to which a correlation between the E-score and the number of ambiguous nucleotides per plastid genome can be generalized to sequencing evenness and assembly quality depends on the utility of the ambiguous nucleotide count as assembly quality indicator. Numerous assembly quality measures have been developed^102–108^ and are typically subdivided into alignment- and sequence-based indicators^109^. Alignment-based quality indicators assess the nucleotide or annotation synteny between genomes and apply to partial genome sequences; the number of ambiguous nucleotides per genome is one such indicator and has been used in various genomic studies^26,110,111^. Sequence-based indicators assess sequence features such as genome length and contiguity and are sensitive to genome completeness but often agnostic to genome structure^104,109^; the number of IR sequence mismatches is one such indicator and used in various IR identification software tools^3,112^. Since both indicators may be sensitive to underexplored factors (e.g., plastidial heteroplasmy)^113^, further research is needed to evaluate their utility as assembly quality metrics^34^. Further research is also needed to confirm that read normalization genuinely improves plastome assembly quality, even if our results provide preliminary evidence to that effect.

The results of this study also indicate that differences in sequencing evenness between sequencing platforms are unlikely due to chance: the E-score differed significantly across plastome assemblies from various Illumina platforms (Table 3). Post-hoc tests confirmed significant differences in multiple pairwise comparisons, often with moderate-to-large effect sizes, especially involving the platforms NextSeq 500, HiSeq 1500 and HiSeq 2000 (Supplementary Table S3). The choice of sequencing platform, specifically the platform HiSeq 2000, was also a strong predictor for sequencing evenness in the linear regression analyses (Supplementary Tables S5). Even the regression tree models confirmed that specific platforms were consistently associated with lower sequencing evenness at top-level splits, regardless of covariate inclusion (Fig. 4D). These differences in sequencing evenness may stem from varying error rates among the platforms^35,114^, which can manifest as sequencing depth variations^115^, as reported in multiple plastid genomic studies^116–118^. Due to our strict sample size and outlier criteria, only a subset of the commonly used NGS platforms was compared: many of the plastid genomes evaluated here were generated between 2009 and 2019 and, thus, primarily reflect first-generation short-read Illumina sequencers; more efficient NGS platforms have since been developed^119^. The results of this study are nonetheless relevant for plastid genomics, as they show for the first time that different NGS platforms can significantly affect sequencing evenness. Future studies on sequencing evenness should assess coverage across newer NGS platforms. Since some covariates in our models – such as partition length and average read length – matched or exceeded platform choice in explanatory power (Fig. 4B), future work should also explore the predictive value of additional, underexplored methodological factors.

The results of our statistical analyses also indicate that differences in sequencing evenness across assembly software are likely due to chance: the E-score did not differ significantly across plastome assemblies generated by different assembly software (Table 3), and the E-score comparisons across select genome assemblies of Freudenthal et al.^9^ indicated no evenness bias from assembly software choice (Fig. 6A). However, previous studies evaluating the impact of assembly software on plastome accuracy and sequencing coverage reached different conclusions: considerable differences in the number, quality, and depth of sequencing coverage of plastome contigs were reported across different assembly software^10,30^. One explanation for this discrepancy is that software choice can affect plastome assembly outcomes, including sequencing depth^120^, but not coverage evenness. Another explanation is that the lack of significant differences across assembly tools reflects limited data, as only a quarter of plastid genomes included software information. Further research is needed to clarify the impact of assembly software choice on sequencing evenness.

In summary, this study indicates that many plastid genomes archived on NCBI Nucleotide are based on sequence data with substantial variation in sequencing depth and evenness, and that this variation is likely influenced by genome structure, sequencing and assembly methodology, and additional, lesser explored factors. In particular, our analyses suggest associations between sequencing coverage and the quadripartite structure, the coding/non-coding subdivision, the number of ambiguous nucleotides, and the used sequencing platform of the plastid genomes, as each of these factors was found to significantly impact coverage. However, the inclusion of covariates in our regression models indicated that additional factors may have equally strong, if not greater, explanatory power for coverage variation. Moreover, no indications of bias in sequencing evenness due to phylogenetic signal or assembly software choice, and only weak indications of phylogenetic bias among the assembly quality metrics were detected, suggesting that the identified results represent genuine patterns. Furthermore, normalizing the distribution of the input sequence data before plastome assembly was found to be a potentially effective strategy to improve the accuracy of plastome assembies. As one of the first studies examining sequencing depth and evenness across structurally and taxonomically diverse plastid genomes, this work provides a baseline for plastome coverage evenness and an initial evaluation of coverage metrics as indicators of plastome quality. Future studies should investigate the typical variation in sequencing evenness across a broader range of plastid genomes and examine the influence of additional factors, especially GC content, intracellular gene transfer, and sequence read normalization.

## Supplementary Material

Supplementary Files

This is a list of supplementary les associated with this preprint. Click to download.

• JenkeEtAlManuscriptrevisedSupplMaterials.pdf

## Figures and Tables

**Table 1.** Overview of the sequencing platforms, assembly software, and average E-scores of the analyzed plastid genomes.

Variable	State	n	%	Σ %	Outl.	E-score	

						$\overset{-}{x}$	s

Seq. platform	GA II	6	3.2	3.2	0	0.89	0.05
	HiSeq 1500	6	3.2	6.5	0	0.95	0.02
	HiSeq 2000	41	22.0	28.5	3	0.93	0.03
	HiSeq 2500	44	23.7	52.2	1	0.94	0.03
	HiSeq 4000	19	10.2	62.4	0	0.91	0.03
	HiSeq X Ten	5	2.7	65.1	0	0.90	0.06
	MiSeq	52	28.0	93.0	3	0.92	0.04
	NextSeq 500	5	2.7	95.7	1	0.89	0.03
	missing	8	4.3	100	-	0.84	0.09

Asm. software	CLC	14	7.4	7.4	1	0.93	0.03
	SOAPdenovo	7	3.7	11.2	1	0.93	0.02
	Velvet	19	10.1	21.3	0	0.92	0.04
	YASRA	5	2.7	23.9	1	0.92	0.03
	missing	146	76.4	100	-	0.90	0.12

Displayed are the names and relative frequencies of the sequencing platforms and assembly software, along with the average E-score ($\overset{-}{x}$) and standard deviation (**s**) for each group. The prefix “Illumina” was omitted to avoid redundancy. Abbreviations: Asm. = Assembly; GA. = Genome Analyzer; Outl. = Outlier; Seq. = Sequencing.

**Table 2.** Table summarizing the data dispersion of the analyzed variables.

	Variable	n	Na	Outl.	min.–max.	q_1_–q_3_	Median	$\overset{-}{x}$	s

Seq. depth	WRSD LSC	161	28	5	0.22–0.78	0.51–0.64	0.59	0.57	0.10
	WRSD IR_B_	163	27	4	0.00–1.19	0.43–0.80	0.64	0.63	0.25
	WRSD SSC	162	28	4	0.11–1.03	0.52–0.72	0.61	0.60	0.17
	WRSD IR_A_	163	29	2	0.00–1.91	0.31–0.72	0.52	0.53	0.28
	WRSD Cod.	188	0	6	0.01–0.98	0.47–0.62	0.54	0.55	0.14
	WRSD Non-cod.	191	0	3	0.00–1.21	0.31–0.70	0.49	0.52	0.25
Seq. evenness	E-score	194	0	-	0.03–0.98	0.90–0.95	0.93	0.90	0.12
Asm. quality	Ambiguous nucl.	194	0	-	0–551	0–0	0	6.43	44.6
	IR mismatches	164	30	-	0–12830	0–0	0	81.6	1001.9

Listed (from top to bottom) are the statistical dispersions of the WRSD counts for the four structural and the coding and non-coding plastome partitions, of the E-score, and of the assembly quality metrics, each calculated across all plastid genomes analyzed. Column ‘Na’ indicates the number of genomes with insufficiently annotated partitions, precluding WRSD and IR mismatch inference. Columns ‘min.–max.’ and ‘q_1_–q_3_’ present value ranges. Abbreviations: Cod. = Coding; Nucl. = Nucleotides. All other abbreviations follow Table 1.

**Table 3.** Kruskal-Wallis test results regarding differences in sequencing depth and evenness.

	Variable	n	p	*η* ^2^

Seq. depth	WRSD/partition	649	<0.001 ***	0.030 ^s^
Seq. evenness	Seq. platform	178	<0.001 ***	0.120 ^m^
	Asm. software	45	0.729	−0.041

Displayed are the sample size, p-value, and effect size for each test. Significance indicators:

* *p* < 0.05

** *p* < 0.01

*** *p* < 0.001.

Effect size indicators:

s small

m moderate

l large.

Abbreviations follow Table 2.

**Table 4.** Pairwise Wilcoxon rank-sum test results regarding differences in sequencing depth.

State 1	State 2	n1	n2	p.adj	*d_c_*

WRSD IR_A_	WRSD IR_B_	163	163	0.002 **	0.413 ^s^
WRSD IR_A_	WRSD LSC	163	161	0.030 *	0.252 ^s^
WRSD IR_A_	WRSD SSC	163	162	0.003 **	0.367 ^s^
WRSD IR_B_	WRSD LSC	163	161	0.008 **	0.313 ^s^
WRSD IR_B_	WRSD SSC	163	162	0.229	0.134
WRSD LSC	WRSD SSC	161	162	0.008 **	0.317 ^s^
WRSD Cod.	WRSD Non.	188	191	0.023 *	0.235 ^s^

Displayed are the sample size, p-value, and effect size for each pairwise comparison. Abbreviations follow Table 1, significance and effect size indicators Table 3. All p-values, except for the comparison between coding and non-coding genome sections, were adjusted using the Benjamini-Hochberg correction.

## Data Availability

The data sets analysed during the current study are available on Zenodo (https://zenodo.org/) under DOIs 10.5281/zenodo.11322182 (https://doi.org/10.5281/zenodo.11322182) and 10.5281/zenodo.15553426 (https://doi.org/10.5281/zenodo.15553426).
